# Light-Induced Transcription in Zebrafish Targets Mitochondrial Function and Heme Metabolism

**DOI:** 10.3390/antiox14101151

**Published:** 2025-09-23

**Authors:** Alessandra Boiti, Hanna T. Weber, Yuhang Hong, Rima Siauciunaite, Sebastian G. Gornik, Nicholas S. Foulkes, Daniela Vallone

**Affiliations:** 1 Biocentis, 05100 Terni, Italy; alessandra.boiti@biocentis.com; 2 Medical Faculty Mannheim, University of Heidelberg, 68167 Mannheim, Germany; hanna.weber@medma.uni-heidelberg.de; 3 Key Laboratory of Application of Ecology and Environmental Protection in Plateau Wetland of Sichuan, Xichang University, Xichang 615000, China; yuhang.hong@xcc.edu.cn; 4 Institute of Biological and Chemical Systems—Biological Information Processing (IBCS-BIP), Karlsruhe Institute of Technology (KIT), 76344 Eggenstein-Leopoldshafen, Germany; rima.siauciunaite2@kit.edu; 5 Center for Organismal Studies (COS), University of Heidelberg, 69120 Heidelberg, Germany; sebastian.gornik@cos.uni-heidelberg.de

**Keywords:** zebrafish, cavefish, D-box, transcriptional regulation, vertebrate evolution

## Abstract

In fish cells, light exposure elevates levels of reactive oxygen species (ROS) and stress-activated MAP kinase activity and thereby induces gene transcription. However, we lack a complete understanding of the function and evolution of this regulatory mechanism. Here, we reveal that a set of mitochondrial and heme metabolism genes is transcriptionally induced in zebrafish cells upon exposure to light or elevated ROS. The integrity of D-box and E-box enhancers in these gene promoters is essential for their transcriptional activation. Furthermore, light-induced transcription of mitochondrial and heme metabolism genes is absent in a cell line derived from the blind Somalian cavefish (*Phreatichthys andruzzii*). This fish species has evolved in perpetual darkness and lacks light-dependent circadian and DNA repair responses as well as D-box-mediated and light- and ROS-induced transcription. PAR-bZip transcription factors bind to and activate transcription via the D-box. Cavefish homologs of these factors share extensive homology with their zebrafish counterparts and lack the deletion mutations that characterize other light-dependent genes in this species. These results extend the role of the D-box as a key regulator of light- and ROS-driven transcription in fish, beyond the circadian clock and DNA repair systems, to also encompass metabolic and mitochondrial function.

## 1. Introduction

Sunlight represents a vital environmental factor for most organisms, ranging from prokaryotic algae to highly complex multicellular organisms, and has been shown to affect many physiological processes at the cellular and organismal levels [1,2,3,4,5,6]. For example, the energy in sunlight is efficiently harnessed into chemical energy by the process of photosynthesis, and sunlight represents the most potent entraining signal (*zeitgeber*) for the circadian clock [7,8], enabling organisms to anticipate daily environmental changes and thereby to enhance survival [9,10,11]. However, both the visible and UV wavelengths of sunlight can also damage macromolecules, for example, inducing inheritable covalent modifications in DNA and influencing the structure and function of proteins [1,12,13,14]. Moreover, cells contain many chromophores, such as porphyrins and flavins, which can interact with light, thereby generating reactive oxygen species (ROS). High levels of ROS are toxic, inducing covalent damage in nucleic acids, proteins, and lipids [15,16], as well as influencing the metabolic and redox state of cells [17,18,19].

One key facet of the cellular response to sunlight is light-regulated gene expression. Specifically, the exposure of cells to sunlight triggers the transcription of subsets of genes that are involved in coordinating appropriate cellular responses. However, the molecular mechanisms by which sunlight exposure and its induction of ROS regulate gene expression remain incompletely understood. We have used various fish species, notably the zebrafish (*Danio rerio*), as animal models to explore these mechanisms. Fish cells are remarkable in that they exhibit directly light-entrainable circadian clocks. Direct exposure of explanted fish tissues and even cell lines to light results in entrainment of their peripheral clocks that contrasts with the situation in mammals, which lack direct photoentrainment. Instead, mammals are reliant on the indirect detection of light by specialized retinal ganglion cells in the retina, which express the photopigment melanopsin and indirectly innervate the central clock in the suprachiasmatic nucleus (SCN). Thereby, light entrains the SCN clock and, via a complex network of systemic signals, the central clock in turn coordinates the phase of clocks in peripheral tissues. For this reason, cell cultures generated from zebrafish embryos [20] or from fin biopsies collected from adult and juvenile zebrafish [21] represent powerful in vitro models to explore light-responsive molecular mechanisms and how they modulate gene expression [3,22]. Using these cell culture models, we revealed that exposure of cells to visible light or UV light elevates cellular ROS levels that, in turn, activate MAP kinase stress signaling pathways [23]. Among the nuclear targets of this stress signaling pathway are D-box enhancer sequences present in the promoters of clock and DNA repair genes. These enhancers are necessary and sufficient for the upregulation of transcription in response to visible light, UV light, and ROS [4,23,24].

An important fish model that has provided additional insight into the function, as well as the evolution of light-regulated gene expression, is the Somalian blind cavefish *Phreatichthys andruzzii*. This species has evolved for more than two million years in subterranean phreatic layers completely isolated from surface water beneath the Somalian desert and exhibits extreme troglomorphic phenotypes. These include complete eye loss, absence of body pigmentation, and various metabolic adaptations for surviving their nutrient-poor environment [25]. Importantly, these fish also lack directly light entrainable clocks as well as light-dependent DNA damage repair mechanisms [4,24,26]. This absence of light-dependent functionality is associated with the loss of light-induced expression of clock and DNA repair genes [4,24,26]. As in zebrafish, visible light or UV light exposure induces increases in cellular ROS levels and activation of MAP kinase stress signaling pathways in cavefish [23]. Furthermore, the promoters of these two classes of genes have retained functional D-box enhancers in cavefish, indicating that transmission of the light signal is attenuated downstream of the MAPK pathway and thereby does not permit transcriptional activation in this species. Therefore, comparing cell signaling and gene expression in zebrafish and *P. andruzzii* cells provides an opportunity to identify key elements of light-responsive cell signaling pathways and how they have been shaped over the course of evolution.

The proline and acidic amino acid-rich (PAR) family of bZip transcription factors has been proposed as a key player in the activation of gene expression via D-box elements [27,28]. In vertebrates, this family of transcription factors consists of three transcriptional activators [28], TEF (Thyrotroph Embryonic Factor), HLF (Hepatic Leukemia Factor) and DBP (D-box Binding Protein) [29,30,31,32], as well as the transcriptional repressor NFIL3 (Nuclear Factor, Interleukin 3 regulated, also known as E4 Binding Protein 4 or E4BP4) [33], which all bind specifically to D-box enhancer sequences in various homo- and hetero-dimeric combinations. In zebrafish, six PAR activators (*tef-1*, *tef-2*, *hlf-1*, *hlf-2*, *dbp-1,* and *dbp-2*) and six repressors (*nfil3-1a*, *nfil3-1b*, *nfil3-2a*, *nfil3-2b*, *nfil3-3a*, and *nfil3-3b*) [27,34,35] have been identified. The relative contribution of these various factors to visible light, UV, and ROS-regulated transcription remains unclear. In addition, it is unclear whether only clock and DNA repair genes or also other classes of genes are directly regulated by sunlight via the same mechanism. Putative D-box sequences have also been found in a set of genes related to stress responses, heme metabolism, mitochondrial function, and binding to retinol [36]. In mammals, conserved D-box sequences have also been shown to regulate the expression of genes involved in diverse processes, including xenobiotic metabolism, thyroid hormone production, and glucose and lipid metabolism [37,38,39,40,41]. In mammals, D-box has been documented to serve as a key element within the clock output pathway, regulating the rhythmic expression of genes involved in physiology and behavior via the circadian clock. This is not the case in fish, where D-box plays a distinct role in regulating the expression of genes in response to light, the so-called input pathway of the clock.

While much attention has been focused on light-inducible circadian clock and DNA damage repair genes in zebrafish cell lines, we still lack a global view of light-induced gene expression in this cell model and how this has been shaped over the course of evolution. Here, we performed a transcriptome analysis of a zebrafish embryo-derived cell line acutely exposed to light. We reveal light-dependent activation of a set of mitochondrial and heme metabolism genes in this zebrafish cell line, as well as in zebrafish embryos. The transcription of these genes is induced by light via D-box enhancer elements in their promoters, implicating this mechanism in a broader control of light-mediated gene expression in zebrafish. As previously reported for D-box-regulated clock genes and genes involved in DNA damage repair, light exposure failed to induce expression of mitochondrial and heme metabolism genes in cell lines prepared from the blind cavefish *P. andruzzii*. Finally, we cloned each cavefish PAR bZip transcription factor and compared the sequences with their zebrafish counterparts. We reveal an overall high degree of similarity between the cavefish and zebrafish proteins and no evidence for the C-terminal truncations we previously documented in this cavefish for genes with a light-related function.

## 2. Materials and Methods

All experiments were performed according to local regulations for biology safety level 1 at the Karlsruhe Institute of Technology (permission number KITGF.KA.10.02).

### 2.1. Zebrafish Embryos

The wildtype WIK zebrafish strain (WIK, *Danio rerio*) was maintained at 28 °C in water circulation systems, under 14:10 light/dark conditions, and fed twice daily [42] (general license for fish maintenance and breeding: Az.: 35-9185.64/BH KIT IBCS-BIP Karlsruhe Institute of Technology (KIT)). Six-month-old fish were crossed to produce embryos for a subset of our experiments. Crossing was performed according to standard methods [42]. All husbandry and experimental procedures were performed following European Legislation for the Protection of Animals used for Scientific Purposes (Directive 2010/63/EU). Embryos were raised for four days in complete darkness, in 28 °C incubators in E3 medium, with the addition of 200 µM 1-phenyl 2-thiourea (PTU) from 24 hpf onwards. At the beginning of the fourth day (96 hpf), embryos were exposed to blue light for up to 6 h (up to 102 hpf) and sampled during this period of illumination. Therefore, the embryos were sacrificed before the free-feeding stage was reached (120 hpf), at which point ethical board approval is required in Germany.

### 2.2. Cell Culture and Treatments

Zebrafish (PAC-2) [20] and cavefish (EPA) embryo-derived cell lines [23] were cultured in Leibovitz L-15 medium (Gibco - Thermo Fisher (Waltham, MA, USA)) supplemented with 15% or 20% Fetal Bovine Serum (FBS, Gibco), respectively, 100 U/mL of penicillin, 100 µg/mL of streptomycin, and 50 µg/mL of gentamicin (Gibco) in an atmospheric CO_2_, non-humidified incubator at 26 °C. For all experiments, cells were seeded in 96-well, 24-well, or 6-well plates at 3 × 10^4^ cells/well, 8 × 10^4^ cells/well, or 3 × 10^5^ cells/well, respectively. As a proxy for elevated ROS, PAC-2 and EPA cells were treated with 300 µM H_2_O_2_, and in order to eliminate any possible direct effects of light exposure, all cells were maintained in constant darkness during the treatment period. Precise experimental details are described in the Figure Legends and Section 3.

### 2.3. Lighting Conditions

Cell and embryo illumination was performed at a constant temperature using a monochromatic blue LED light source (λpeak = 468 nm, Kopa (Barcelona, Spain)) adjusted to deliver the photon flux (1.42 ± 0.04 × 10^18^ photons/s/m^2^). Precise details of lighting conditions in our experimental analysis are provided in the Figure Legends and Results sections.

### 2.4. RNA Extraction and RT-qPCR Analysis

After two days in darkness, PAC-2 and EPA cells were exposed to up to 6 h of blue light, and samples were collected in Trizol reagent (Thermo Fisher (Waltham, MA USA)) for RNA extraction. Total RNA was extracted according to the manufacturer’s instructions. Total RNA (4 µg) from PAC-2 and EPA cells was shipped to Novogene (UK) for mRNA sequencing and preliminary bioinformatic analysis. For gene expression analysis, reverse transcription was performed with the RevertAid reverse transcription kit (Thermo Fisher) using random primers. SyBrGreen (Promega (Walldorf, Germany)) master mix was used for quantitative PCR analysis in the Real-Time PCR ABI QuantStudio3 qPCR Cycler (Thermo Fisher). Primers are shown in Supplementary Table S1. Data were analyzed according to the 2^−ΔΔCT^ method, and expression of *β*-actin was used for normalization.

### 2.5. RNA-Seq Analysis

**Figure 1:**
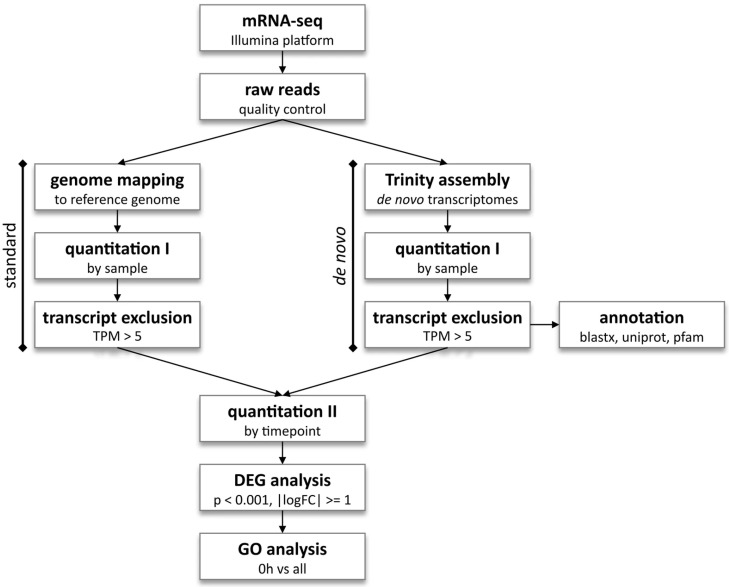
mRNA-sequencing analysis pipeline. The zebrafish *D. rerio* dataset, for which a reference genome is available, was subjected to standard transcriptome sequencing analysis (left), as well as de novo analysis (right). Both zebrafish and cavefish *P. andruzzii* datasets were subjected to de novo analysis with Trinity software, as no reference genome is available. Quantitation, DEG, and GO analyses and graphs were performed with R software version 4.3.2.

Sequencing using the Illumina platform, as well as subsequent quality control (error rate distribution, GC-content distribution, and data filtering), mapping to the reference genome (GRCz11 for the *D. rerio* dataset), and gene expression quantification were performed by Novogene UK. Since there is no available reference genome for the cavefish *P. andruzzii*, de novo transcriptome analysis was performed for both species. Briefly, Trinity software version 2.13.2 [43] was used to assemble the short reads into full transcripts, which were then quantified and annotated via alignment of the transcripts and the longest Open Reading Frame (ORF) against UniProt via Blast-X version 2.13 alignment. Domains were predicted via alignment in the PFAM database. Transcripts within the datasets were quantified and excluded based on the Transcript Per Million (TPM) cut-off of 5. For all analyses, transcripts were quantified for each sample, and Differential Expression analysis was performed across all timepoints, considering transcripts with |logFoldChange| >= 1 and adjusted *p*-value < 0.001 to be differentially expressed. Finally, functional enrichment testing was performed via Gene Ontology (GO) analysis on 0 h vs. 6 h Differentially Expressed Genes (DEGs). A detailed pipeline for the analyses is shown in Figure 1.

### 2.6. mRNA Stability Assay

Actinomycin-D was used as an inhibitor of cellular transcription. PAC-2 cells were treated with 5 µg/mL Actinomycin-D (A1410, Sigma-Aldrich (St. Louis, MO, USA)) before exposure to blue light (t = 0 h), and samples were collected in Trizol at 1, 3, and 6 h. Controls treated with Actinomycin-D were kept in darkness. Following RNA extraction and cDNA synthesis, RT-qPCR was used to measure the relative amounts of mRNA present in the samples. Primers used are listed in Supplementary Table S1.

### 2.7. Promoter Bioinformatic Analysis

Promoter region sequences, 1 kb upstream of the Transcription Start Site (TSS) plus the 5′ untranslated regions of genes, were retrieved using BioMart from Ensembl release 109 (GRCz11). Clover [44] was used to screen the promoters for putative D-box and E-box sequences. Putative light-responsive promoter fragments containing D-box-like and E-box-like motifs were cloned into the pGL3Basic (Promega) luciferase expression vector. A smaller fragment of *hebp2* (144 bp) was cloned into the minimal promoter luciferase expression vector pLucMCS (Stratagene (La Jolla, CA, USA)). The sequences of single, predicted D-box and E-box sequences were mutated (4–6 bp each) using the Q5^®^ Site-Directed Mutagenesis kit (Promega) with the aim of disrupting the enhancer function.

### 2.8. P. andruzzii PAR-bZip Transcription Factor Cloning

Rapid Amplification of cDNA Ends (RACE) PCR (SMARTer RACE 5′/3′ Kit, Takara Bio (San Jose, CA, USA)) was used to amplify the 5′ and 3′ ends of the HLF-1, TEF-2, and Nfil3-1b sequences of the Somalian cavefish according to the manufacturer’s instructions. The sequences for the other PAR-bZip and Nfil3 transcription factors were obtained from a partial genome sequencing dataset after confirmation via alignment with the zebrafish counterparts. The complete mRNA coding sequences were amplified from EPA cell cDNA and cloned into the CMV-driven pCS2-MTK vector for expression in eukaryotic cells. The start codon was mutated so that a 5′ 5x myc-tag sequence (EQKLISEEDL) was incorporated adjacent to the N-terminus of each PAR factor. All cloning was confirmed first by sequencing (Microsynth Seqlab, Göttingen, Germany) and then by their expression in transiently transfected cells via Western blot analysis for the myc-tagged proteins (see Supplementary Figure S2). The zebrafish PAR-bZip and Nfil3 factors studied were cloned previously [34].

### 2.9. Western Blotting

PAC-2 cells in 6-well plates were transfected with 1 µg of the zebrafish and cavefish PAR-bZip expression vectors and kept in darkness for 48 h before lysis with Passive Lysis Buffer (Promega). Samples were run on 10% polyacrylamide-SDS gels together with the Color Prestained Protein Standard, Broad Range (10–250 kDa, New England BioLabs (Ipswich, MA, USA)), and blotted onto Immobilon^®^-P PVDF membrane (Merck Millipore, Tullagreen, Ireland). A mouse anti-Myc tag antibody (1:1000, Sigma-Aldrich) and goat anti-mouse polyclonal antibody (1:7500, Sigma) were used to detect protein expression. Visualization was performed by using the ECL detection system (Bio-Rad, (Hercules, CA, USA)). Images were acquired and analyzed by using Image Lab Software version 6.1.0 build 7 (Bio-Rad). Western blot data are presented in Supplementary Figure S2.

### 2.10. Cell Transfection and Bioluminescence Assays

Transfection was performed 24 h following cell seeding using FuGene HD (Promega), as described elsewhere [4,23,24]. For in vivo bioluminescence assays, 25–100 ng of the luciferase reporter plasmid was transfected into each well of a 96-well plate. After 24 h, 0.5 mM D-luciferin Firefly potassium salt (FL08607, Biosynth, (Bratislava, Slovakia)) was added to the culture medium, and bioluminescence was measured each hour using Topcount NXT or Envision counters (Perkin Elmer (Shelton, CT, USA)). For in vitro experiments, cells were transfected with 50–200 ng of the luciferase reporter vector, 50 ng of the *β*-galactosidase expression vector (pcDNA3.1/myc-His/lacZ, Invitrogen (Carlsbad, CA, USA)), and 1 ng of the pCS2-MTK expression vector containing the transcription factor of interest and lysed after 48 h using Firefly Lysis Buffer. The cell lysates were used for luciferase assays, and a *β*-galactosidase assay served as a control for transfection efficiency normalization. All transiently transfected fish cell culture experiments and standard molecular cloning methodology were performed according to local regulations for biology safety level 1 at Karlsruhe Institute of Technology (Permission number KITGF.KA.10.02)).

### 2.11. Statistical Analysis

Data were analyzed and graphs were produced using R software version 4.3.2 and InkScape version 1.4.2. All results are expressed as means ± Standard Error of the Mean (SEM). ANOVA with multiple comparisons post hoc tests (Tukey HSD) or Bonferroni corrections were used to determine significance. Values of *p* < 0.05 were considered to be statistically significant, and values of *p* < 0.05, *p* < 0.001, and *p* < 0.001 are represented in graphs by *, **, and ***, respectively. Detailed statistical information and the data used to make the figures are reported in Supplementary Dataset S1.

## 3. Results

### 3.1. The Light-Induced Transcriptome in Zebrafish Cells

**Figure 2:**
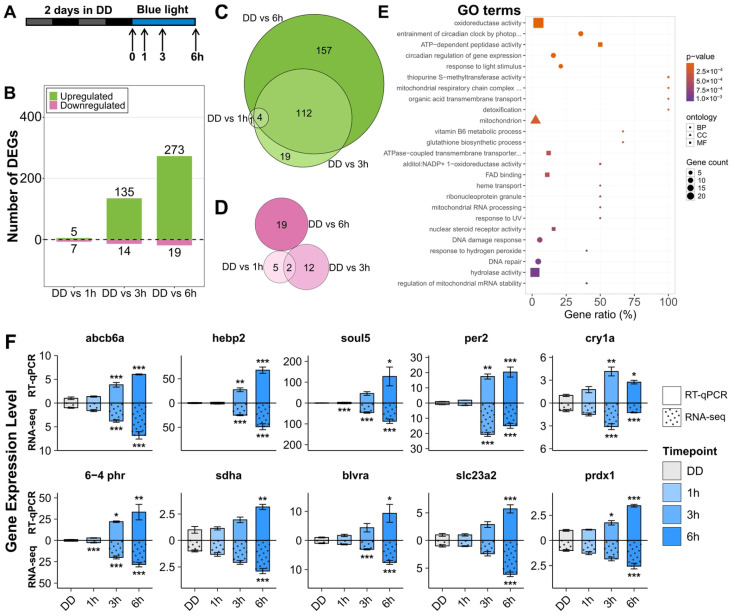
Transcriptome of zebrafish PAC-2 cells exposed to blue light. (**A**) Experimental design. Cells were initially maintained in darkness for two days to dampen clock- and light-dependent gene expression before exposure to 468 nm blue light for up to six hours. Following RNA extraction at the indicated timepoints and subsequent sequencing, the RNA-seq data were analyzed according to the “standard” pipeline represented in Figure 1. (**B**) Number of significantly up- and downregulated genes at 1 h, 3 h, and 6 h compared to unexposed samples (0 h, DD). (**C**,**D**) Venn diagrams show significantly upregulated (293 genes, (**C**)) and downregulated (38 genes, (**D**)) genes among the three comparison groups. (**E**) Top 25 enriched GO terms for DEGs at 6 h vs. DD in PAC-2 cells. The percentage of upregulated genes within their specific category is indicated on the *x*-axis. Size indicates the number of upregulated genes for each term; shape indicates the ontology (BP: biological process; CC: cellular component; MF: molecular function), and color indicates the adjusted *p*-value from high (blue) to low (red). (**F**) RT-qPCR validation of blue light-induced expression of selected upregulated genes. In each graph, RT-qPCR data are plotted above the *x*-axis, and RNA-seq data are below (dotted bars). Values are represented by mean ± SEM of fold induction (*n* = 3). One-way ANOVA followed by Tukey’s HSD post hoc multiple comparison tests against the DD control results can be found in Supplementary Dataset S1. *p* < 0.5, *p* < 0.01, and *p* < 0.001 are represented by *, **, and ***, respectively.

In order to obtain a view of the full scope and, ultimately, the functional significance of light-induced gene expression in zebrafish cells, we initially characterized the light-induced transcriptome in the PAC-2 zebrafish cell line (originally established from zebrafish embryos [20]). Thus, PAC-2 cells were first kept in complete darkness for two days to ensure significant downregulation of light-induced gene expression as well as a dampening of clock-regulated gene expression, and then they were exposed to one, three, or six hours of Blue Light (BL) prior to RNA extraction. A monochromatic blue light source (λpeak = 468 nm) was chosen based on previous studies demonstrating a robust transcriptional response upon exposure to this wavelength [23]. Furthermore, by sampling at these timepoints, elevated mRNA levels were detected for all previously characterized light-regulated genes [4,23,45]. A control sample not exposed to blue light was harvested as time 0 (dark control, DD) (see the experimental scheme in Figure 2A). RNA extracted from the cell samples was subjected to quality control assurance (Nanodrop and Agilent 5400 assay systems) before sequencing and performing bioinformatic analysis. The resulting dataset can be downloaded from GEO (GSE290582).

Differentially Expressed Genes (DEGs) in zebrafish cells were identified as having a |log_2_FoldChange| > 1 and *p*-value adjusted for multiple comparisons *p*-adj < 0.001. A total of 331 genes were differentially expressed after either 1, 3, or 6 h of blue light exposure compared to the DD control. Blue light mostly led to the upregulation of genes at 3 and 6 h (Figure 2B–D for up- and downregulated genes, respectively). Gene Ontology (GO) analysis of the genes upregulated at 6 h was performed to categorize the biological processes, cellular compartments, and molecular functions affected by light exposure. The top 25 enriched GO terms are shown in Figure 2E. As expected, the gene set was significantly enriched in the GO terms related to circadian clock regulation and circadian clock entrainment by photoperiod. Other significantly enriched categories included DNA damage response and DNA repair, as well as response to hydrogen peroxide and oxidative stress, detoxification processes, and glutathione biosynthesis. Interestingly, the GO results also revealed significant enrichment of processes related to mitochondria structure and function, as well as to heme biosynthesis and metabolism.

### 3.2. Effect of Light on Mitochondrial and Heme Metabolism Genes

Among the new classes of genes found to be light-regulated were genes involved in the biosynthesis and transport of heme and other porphyrins (*abcb6a*, *blvra*, *fech*, *slc40a1*/*ferroportin1*, *slc48a1a*, *tfr1a*, and *tspo*), as well as genes encoding heme-binding proteins and hemoproteins (*txbas1*, *cyb5a*, *cyp1ae1*, *hebp2*, and *soul5*). Among the upregulated mitochondria-related genes were those coding for subunits of the ETC complexes and their assembly (*sdha*, *sdhb*, *sdhaf1*, *sdhaf2*, *sdhaf3*, *afg3l2*, *chchd4b*, and *ttc19*), as well as genes involved in the regulation of mitochondrial architecture and connectivity (*mff*, *adck1*, *rap1gd1*, *retsat*, and *trf1a*), and those implicated in the regulation of mitochondrial activity and membrane permeability (*hebp2*, *bbc3*, *retsat*, and *sirt4*). A total of 63 significantly upregulated genes belonging to these categories were identified as a group, which will hereafter be referred to as mitochondria and heme-related genes. A detailed list of all genes belonging to this group can be found in Supplementary Dataset S1. To validate our RNA-seq results, a subset of genes was selected based on the strength of induction and their established relevance [46,47,48,49,50]. Their induced expression, together with that of light-regulated controls, clock genes (*per2* and *cry1a*), and a DNA-repair gene (*6-4phr*), was confirmed via RT-qPCR. Figure 2F represents the RT-qPCR results compared with data obtained by RNA-seq analysis. These results are also consistent with no significant changes in the expression of light-responsive genes occurring in the cell cultures under DD conditions during the 6 h sampling period for the RNA-seq and RT-qPCR analysis (see Figure S1).

### 3.3. Comparative Analysis of Light-Mediated Gene Expression in Zebrafish and Cavefish Cells

In the Somalian cavefish *(P. andruzzii*), we previously reported that light/ROS-induced expression of clock genes and DNA-repair genes is absent [4,23,24,26]. Does this pattern extend to all other light-induced transcripts identified in the zebrafish cell line? Providing answers to this question allows us to assess whether the D-box control mechanism serves as a global regulator of light and ROS-induced gene expression. Thus, as the next step, we compared the light-induced zebrafish transcriptome with RNA-seq data from EPA cells (a cell line previously established from *P. andruzzii* embryos [23]) using the same experimental design.

**Figure 3:**
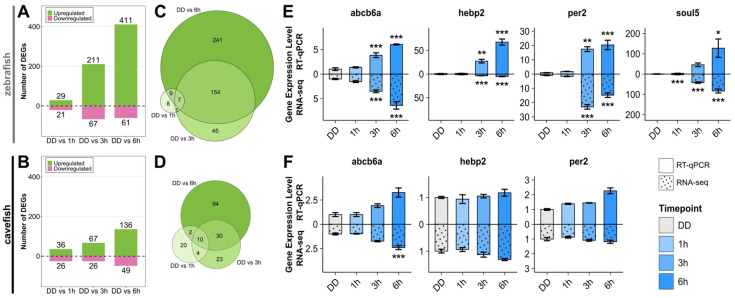
Transcriptome of zebrafish PAC-2 (top) and cavefish EPA (bottom) cell lines exposed to blue light. (**A**,**B**) Number of significantly up- and downregulated genes at 1 h, 3 h, and 6 h, compared to nonexposed samples (0 h, DD) for zebrafish (**A**) and cavefish (**B**) cells following the de novo pipeline represented in Figure 1. (**C**,**D**) Venn diagrams show significantly upregulated genes among the three comparison groups for zebrafish (469 genes) (**C**) and cavefish (183 genes) cells (**D**). (**E**,**F**) mRNA sequencing and RT-qPCR validation of blue light-induced expression of selected upregulated genes in zebrafish (**E**) and cavefish (**F**) cells. In each graph, RT-qPCR data are plotted above the *x*-axis and RNA-seq data below (dotted bars). Values are represented by mean ± SEM of fold induction (*n* = 3). One-way ANOVA followed by Tukey’s HSD post hoc multiple comparison tests against the DD control results can be found in Supplementary Dataset S1. *p* < 0.5, *p* < 0.01, and *p* < 0.001 are represented by *, **, and ***, respectively.

To date, a complete annotated genome sequence is not available for *P. andruzzii.* Therefore, the transcriptome sequences of both the cavefish and zebrafish cell lines were initially assembled with a de novo pipeline before comparison and analysis (Figure 1). DEGs were identified with the same thresholds as the zebrafish dataset. The analysis showed that the magnitude of transcriptional changes occurring in response to blue light in cavefish cells was much smaller than in zebrafish cells. In the PAC-2 cells, a total of 607 DEGs were identified at 1, 3, or 6 h of exposure to blue light compared to the DD control, while only 278 DEGs overall were identified in cavefish. For both species, the number of identified DEGs increased with increasing exposure to blue light, with few genes being up- or downregulated after 1 h of exposure (Figure 3A,B). Furthermore, zebrafish cells displayed a more sustained response to light, with 154 genes (33% of all upregulated genes) consistently upregulated after both 3 and 6 h of exposure (Figure 3C). In contrast, in the transcriptomic response to light of cavefish cells, only 30 genes were upregulated (16% of all upregulated genes) at both 3 and 6 h, suggesting a more transient pattern of gene upregulation (Figure 3D).

While the GO analysis indicated significant enrichment of mitochondrial and heme-related processes in the zebrafish dataset, no such enrichment was observed in the cavefish dataset (Supplementary Dataset S1). GO terms related to circadian clock entrainment, response to light, and DNA repair were also not significantly enriched in cavefish. The expression of selected genes from the mitochondrial and heme-related gene pool was confirmed with RT-qPCR for both species (Figure 3E,F). Consistently reflecting the RNA-seq findings, all genes except *abcb6a* were significantly upregulated in zebrafish but not cavefish cells. *abcb6a* was upregulated in both cell types according to both RNA-seq and RT-qPCR analyses.

### 3.4. Induction of Mitochondria and Heme-Related Genes by Elevated ROS Levels

**Figure 4:**
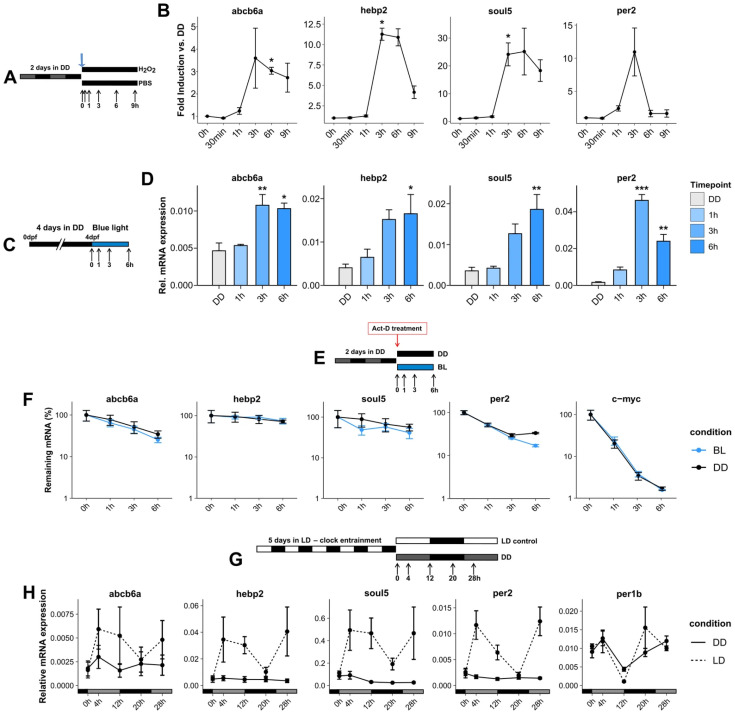
Upregulation of mitochondrial and heme genes is an organism-wide response, dependent on de novo transcription and clock-independent. (**A**) Experimental design for exposure of PAC-2 cells to H_2_O_2_. PAC-2 cells were kept in darkness for two consecutive days, followed by treatment with 300 μM H_2_O_2_, and were then maintained in darkness until collection at timepoints indicated as hours (h) following the addition of H_2_O_2_ to the cultures. A set of cells treated with PBS was sampled in parallel in darkness in order to control for any time-dependent changes in gene expression in the cell cultures that might occur independently of exposure to ROS. (**B**) RT-qPCR of ROS-induced expression of selected upregulated genes in PAC-2 cells. Values are represented by mean ± SEM (N = 3) of fold induction of treatment vs. control. One-way ANOVA followed by Tukey’s HSD post hoc multiple comparison tests was performed against the control results. (**C**) Experimental design for blue light exposure of 4 dpf zebrafish embryos. Zebrafish embryos were raised for 96 h in complete darkness, followed by exposure to 468 nm blue light for up to six hours. During illumination, samples were harvested periodically at timepoints indicated as hours (h) following the start of exposure to blue light. (**D**) Relative mRNA expression of *abcb6a*, *hebp2*, *soul5*, and *per2* in response to blue light in zebrafish embryos. Values are represented by mean ± SEM (N = 3). One-way ANOVA followed by Tukey’s HSD post hoc multiple comparison tests was performed against the DD control results. *p* < 0.5, *p* < 0.01, and *p* < 0.001 are represented by *, **, and ***, respectively. (**E**) Experimental design for mRNA stability of genes following blue light exposure. Zebrafish cells were treated with Actinomycin-D (5 µg/mL) before exposure. During illumination, samples were harvested periodically at timepoints indicated as hours (h) following the start of the combined Actinomycin-D treatment and exposure to blue light. (**F**) RT-qPCR analysis of *abcb6a*, *hebp2*, *soul5*, and *per2*, under blue light (blue) and in darkness (black). *c-myc* expression is measured to serve as a positive control for Actinomycin-D treatment. Data are plotted as the percentage (mean) of remaining mRNA ± SEM (N = 3) compared to cells not treated with actinomycin-D (0 h) on the *y*-axis. Expression levels in the two lighting conditions and across timepoints are compared via two-way ANOVA. (**G**) Experimental design for assaying circadian clock regulation. Zebrafish cells were entrained to 12 h:12 h light:dark cycles (LD) for five days, followed by one more day in LD or complete darkness (DD). Samples were harvested periodically at the indicated timepoints, in hours (h), following the start of the final LD cycle or the transfer to DD conditions. (**H**) RT-qPCR analysis of *abcb6a*, *hebp2*, and *soul5*, as well as *per2*, a light-regulated clock gene, and *per1b*, a clock-regulated gene. Relative mRNA expression of cells kept in LD (dashed line) or DD at different timepoints (*x*-axis) is plotted on the *y*-axis as mean ± SEM (*n* = 3). Detailed statistical analysis and the data for this figure can be found in Supplementary Dataset S1.

Visible light has been shown to induce ROS production in zebrafish cells, and the D-box enhancer is known to be necessary for the response to ROS, providing one mechanism by which light activates D-box-driven transcription. Previous studies have demonstrated that ROS increases alone significantly upregulate the expression of the D-box-controlled circadian clock and DNA damage repair genes in PAC-2 cells [23,24,45]. To test whether this also applies to *abcb6a*, *hbp2*, and *soul5*, PAC-2 cells were treated with 300 μM hydrogen peroxide, and gene expression was assessed via RT-qPCR compared with controls kept in constant darkness (Figure 4A). The treatment led to the upregulation of all three genes after 3, 6, and 9 h of treatment (Figure 4B).

### 3.5. Transcriptional Activation of Mitochondria and Heme-Related Genes via Blue Light In Vivo

Our transcriptomic and RT-qPCR gene expression analyses revealed that blue light upregulates the expression of various functional classes of genes in zebrafish cells, but not in cavefish cells. The light-induced expression pattern of the mitochondria and heme-related genes closely resembles that of light-responsive circadian clock and DNA-repair genes, whose expression has previously been documented in vivo in the context of the zebrafish larva. Thus, we examined whether this is also the case for this class of mitochondria and heme genes. We illuminated 4 dpf zebrafish embryos with blue light for up to 6 h and collected samples to perform RT-qPCR (Figure 4C). Our choice of this zebrafish developmental stage was based on the documented emergence of a functional circadian clock and light-regulated clock gene expression by 4 dpf [51]. The blue light exposure led to a significant increase in the expression of the *abcb6a*, *hebp2*, and *soul5* genes, as observed for the clock gene *per2* used as a positive control (Figure 4D). This pattern in zebrafish embryos mirrored the results seen in PAC-2 cells and is consistent with previous reports for circadian clock and DNA repair genes [36].

Is this increase in RNA expression in response to light purely due to transcriptional regulation, as for the clock and DNA repair genes? To address this question, we initially tested whether the mRNA stability of the mitochondrial and heme-related genes changed under blue light exposure. PAC-2 cells were treated with Actinomycin-D (5 µg/mL), an inhibitor of de novo gene transcription, immediately before exposure to 1, 3, or 6 h of blue light or darkness as a control. This experimental approach blocks any transcription activation and tests for any changes in mRNA stability (Figure 4E). *c-myc* mRNA levels were monitored as a positive control since this transcript is well documented to have a high turnover [52]. This treatment resulted in no significant difference in degradation time for all the mRNAs analyzed between the samples maintained in DD and the samples exposed to blue light. This indicates that the increased expression of these genes is the result of light-mediated transcriptional activation (Figure 4F).

### 3.6. Lack of Circadian Clock Regulation for the Light-Activated Mitochondrial and Heme Genes

It has previously been shown that the expression of many light-induced genes also oscillates in a circadian manner due to the presence in their promoters of canonical or non-canonical circadian enhancer elements termed E-boxes [27]. Therefore, rhythmic expression of these genes persists for several cycles when transferred to constant darkness. We next tested whether this was also the case for the mitochondrial and heme-related genes. PAC-2 cells were entrained to 12 h:12 h light:dark cycles (LD) for five consecutive days, followed by transfer to constant darkness or LD conditions once again, and gene expression was measured at regular intervals over a 28 h period (Figure 4G). The expression of the *per1b* gene, which exhibits similar oscillations in both LD and DD conditions, was used as a positive control for a purely circadian clock-regulated gene. Instead, the expression of the *per2* gene, which exhibits oscillation only under LD conditions, was used as a positive control for a purely light-responsive gene. Our results showed that *abcb6a*, *hebp2*, and *soul5* all behave as exclusively light- and not circadian clock-regulated genes (Figure 4H).

### 3.7. D-Box Enhancer Elements Regulate the Light and ROS Response of Mitochondria and Heme-Related Genes

**Figure 5:**
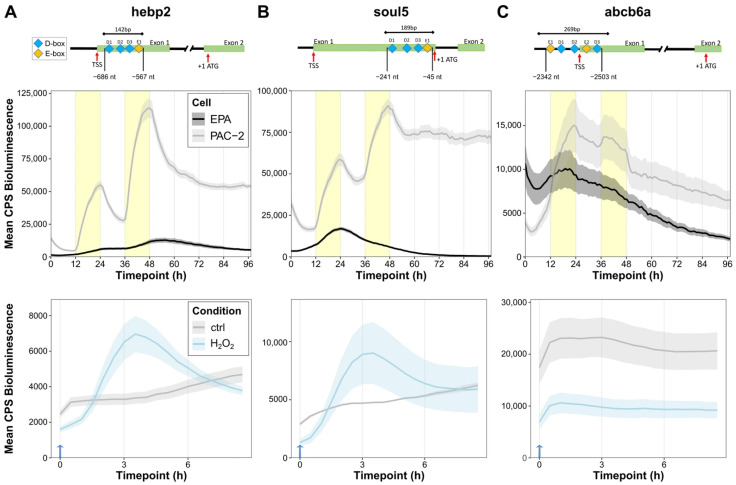
The promoters of mitochondrial and heme-related genes have light- and ROS-responsive modules. (**A**–**C**) Above: Bioinformatic prediction of D-box (blue) and E-box (yellow) sequences in the promoters of the hebp2 (**A**), abcb6a (**B**), and soul5 (**C**) genes. Exons, Transcription Start Sites (TSS), and start codons (ATG) are indicated for each promoter fragment. The upper bars indicate the fragments cloned in luciferase reporter vectors and their respective lengths in base pairs (bp). Distances from the ATG are also indicated. Middle: Representative (of 3) in vivo bioluminescence assays in PAC-2 (gray) and EPA (black) cells transfected with the hebp2-Luc (**A**), abcb6a-Luc (**B**), and soul5-Luc (**C**) luciferase reporters exposed to 24 h of darkness followed by LD cycles. Below: Representative (of 3) in vivo bioluminescence assays in PAC-2 cells treated with H_2_O_2_ after transfection with the hebp2-Luc (**A**), abcb6a-Luc (**B**), and soul5-Luc (**C**) luciferase reporters and kept in complete darkness. Traces from cells treated with 1 mM H_2_O_2_ at 0 h (blue arrow) are plotted in light blue; untreated controls are plotted in gray.

Which mechanism drives the light-dependent activation of *abcb6a*, *hebp2*, and *soul5* gene expression? The findings above resemble the response to light of *per2* and *6-4phr* expression, which is the result of light-mediated transcriptional activation via D-box enhancer elements [4,27]. Therefore, as a first step, we performed a bioinformatic promoter analysis with Clover software (release date 2012-02-16) [44] to test for the presence of D-box enhancers in the promoters of our 63 genes of interest. The software predicted putative D-box sequences within the 5′ Untranslated Region (UTR) and 1 kb upstream of the Transcription Start Site (TSS) in 70% of mitochondria and heme-related genes (the results are shown in Supplementary Dataset S1). In addition, putative E-box sequences were predicted in 48% of these genes. In the case of our three selected genes, three putative D-box and one E-Box sequences were identified within the promoters of *hebp2 (*Figure 5A, above) and *soul5* (Figure 5B, above), while three D-box and two E-box sequences were identified in the *abcb6a* promoter (Figure 5C, above). Indeed, the presence of E-box-like sequences in the proximity of D-boxes is consistent with cooperation between these two enhancer elements to mediate light-induced expression, as we previously reported for the regulation of *per2* gene expression [27,45].

To directly test the contribution of these enhancer elements to directing light-regulated transcription of these genes, fragments of each promoter, encompassing the putative light-responsive promoter sequences, were cloned into a luciferase reporter expression vector, hereafter referred to as hebp2-Luc, abcb6a-Luc, and soul5-Luc. The constructs were then transfected into light-responsive PAC-2 zebrafish cells, as well as into non-light-responsive cavefish EPA cells, and then monitored for their luciferase expression during two days under LD cycles followed by transfer to two days in complete darkness (DD) (Figure 5A–C, middle). All three promoters showed robust light-induced bioluminescence during LD in zebrafish PAC-2 cells that was not maintained under DD conditions, indicating that our promoter constructs included the regulatory sequences required for the light responsive transcription observed with the endogenous genes. Instead, transfection of all three bioluminescent reporters in EPA cells did not elicit a rhythm of bioluminescence in either LD or DD conditions. In addition, consistent with our previously published results regarding D-box-regulated expression of the *6-4phr* gene, the three bioluminescence promoter reporters transfected in zebrafish cells were also induced following H_2_O_2_ treatment (Figure 5A–C, below). This again mirrors endogenous mRNA expression of these genes (Figure 4B), as observed for the promoters of the *cry1a* and *per2* genes [23,45].

**Figure 6:**
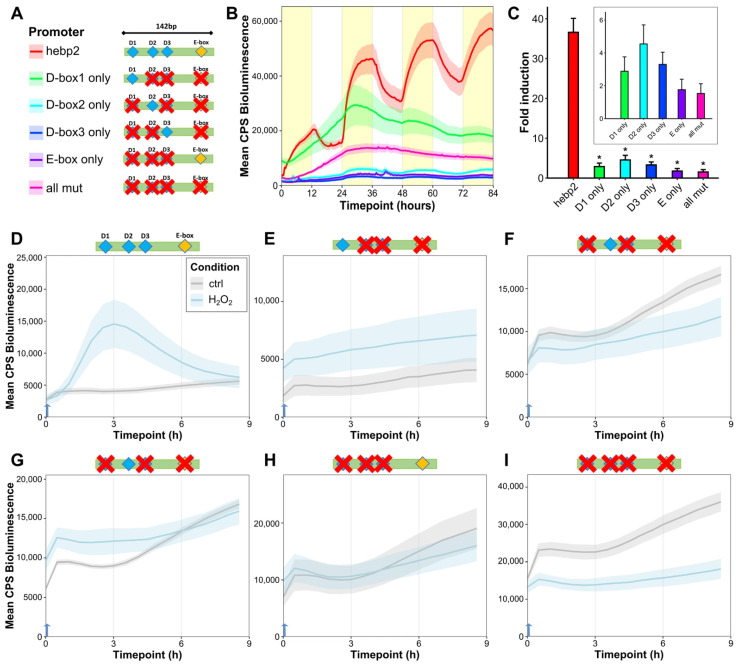
D-box enhancers mediate light- and ROS-responsiveness of promoters of mitochondrial and heme-related genes. (**A**) Schematic representation of hebp2-Luc mutated constructs. The promoter fragment was mutated (indicated by red crosses) so only one predicted D-box or E-box element (or none) was retained. (**B**) Representative (of 3) in vivo bioluminescence assay in PAC-2 cells transfected with hebp2-Luc and its mutant constructs exposed to LD cycles. Mean bioluminescence (CPS) as a function of time (hours) is plotted. Lines show mean ± SEM across N = 6 wells. Lights-on periods are indicated by the yellow boxes. (**C**) In vitro bioluminescence assay of PAC-2 cells transfected with hebp2-Luc and its mutant constructs following 8 h exposure to blue light. Fold induction of relative bioluminescence levels compared with controls kept in darkness at a single timepoint, ± SEM (*n* = 3), is plotted on the *y*-axis. The β–galactosidase assay was used to normalize for transfection efficiency. Differences between the unmutated hebp2-Luc construct and each of the mutant constructs are compared via pairwise *t*-tests with Bonferroni correction. Detailed statistical analysis can be found in Supplementary Dataset S1. *p* < 0.5, *p* < 0.01, and *p* < 0.001 are represented by *, **, and *** respectively. (**D**–**I**) In vivo bioluminescence assays in PAC-2 cells treated with H_2_O_2_ after transfection with hebp2-Luc and its mutant constructs and kept in complete darkness. Traces from cells treated with 1 mM H_2_O_2_ starting at 0 h (blue arrow) are plotted in light blue; untreated controls are plotted in gray.

To further investigate the specific role of each D-box and E-box sequence in light-mediated gene expression, we systematically mutated each D-box and E-box in the hebp2-Luc reporter via site-directed mutagenesis, resulting in four new constructs where only a single D-box or E-box was retained. In addition, a control construct where all D-box and E-box sequences were mutated simultaneously was also generated (Figure 6A).

When transfected into PAC-2 cells and exposed to an LD cycle, all the mutated constructs in an in vivo luciferase assay showed attenuated activation in response to light compared to the intact, non-mutated hebp2-Luc reporter (Figure 6B). Treatment with H_2_O_2_ did not activate the mutated constructs (Figure 6D–I). An in vitro luciferase assay of cells transfected with these same constructs and exposed to blue light for eight hours also revealed significantly reduced activation of the mutated promoters measured at a single timepoint (Figure 6C). These assays indicate that the induction of hebp2-Luc by light relies on the function of both D-box and E-box sequences and that a synergistic effect between these enhancers underlies the light- and ROS-dependent induction of *hebp2*.

### 3.8. Zebrafish PAR-bZip Transcription Factors Activate the Hebp2-Luc Reporter

**Figure 7:**
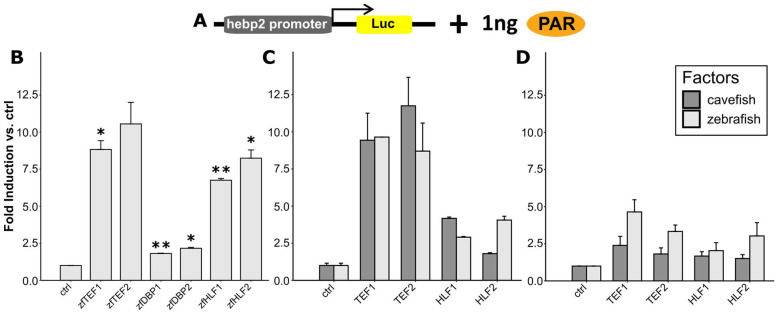
Activation of hebp2-Luc by the PAR transcription factors in PAC-2 and EPA cells. (**A**) Schematic representation of the transfected plasmids: 50 ng hebp2-Luc and 1 ng of the PAR factors expression vectors. The arrowhead denotes the startpoint and direction of transcription from the promoter. (**B**–**D**) In vitro bioluminescence assay results of zebrafish PAC2 cells cotransfected with the hebp2-Luc luciferase reporter and each of the 6 zebrafish PAR factors expression vectors (*n* = 3) (**B**), the zebrafish and cavefish TEF and HLF factors (**C**), and EPA cells transfected with the zebrafish and cavefish TEF and HLF factors (N = 3). Cells were maintained in darkness for 48 h before lysis. Fold induction of relative bioluminescence compared to the control, and mean ± SEM are plotted. A β–galactosidase assay was used to normalize for transfection efficiency. Differences between zebrafish transcription factors and the control are compared via t-tests with Bonferroni correction. Detailed statistical analysis can be found in Supplementary Material 1. *p* < 0.5, *p* < 0.01, and *p* < 0.001 are represented by *, **, and ***, respectively.

Previous reports have demonstrated that PAR-bZip transcription factors bind to the D-box enhancer and regulate the transcription of various light-induced genes, including circadian clock and DNA repair genes [24,26,45,53]. To test whether this is also the case for the *hebp2* promoter, we compared the activation mediated by each zebrafish PAR-bZip homolog [34] in an in vitro luciferase assay using the hebp2-Luc reporter in PAC-2 cells (Figure 7A,B). Our results reveal a range of activation of reporter gene expression, with the strongest activation observed for the TEF and HLF factors. Cavefish-specific loss of PAR-bZip factor function has been proposed as a potential contributor to the loss of light-induced gene expression in *P. andruzzii*. Thus, we amplified the coding sequences of all these *P. andruzzii* factors by RT-qPCR from EPA cell RNA and cloned them into the pCS2-MTK expression vector. The predicted *P. andruzzii* and zebrafish amino acid sequences were aligned, revealing that the C-terminal portions of the PAR-bZip proteins, containing the PAR, basic, and leucine zipper domains, are highly conserved between these species, while the N-terminal sequence of each factor is more divergent (Table 1). Despite the absence of premature truncation mutations that we previously reported in various opsin, clock, and DNA repair genes [4,26], the cavefish factors exhibited non-conservative amino acid substitutions in their N-terminal regions, which could potentially affect their structure and function. Might the amino acid differences observed between these two cyprinid species account for the significant deficiency in light-induced *hebp2* gene expression? To address this question, we initially compared the activation of *hebp2* expression mediated by the zebrafish and *P. andruzzii* homologs of TEF and HLF in transfected PAC-2 cells (Figure 7C). Ectopic expression of the recombinant PAR-bZip factors was initially confirmed by Western blotting analysis of the myc-tagged zebrafish and cavefish proteins (Figure S2).

Our results showed that both the zebrafish and the cavefish factors robustly activated hebp2-Luc reporter expression in PAC-2 cells, with some species-specific differences in the observed amplitude of reporter gene activation (Figure 7C). However, repeating these transfection assays in cavefish EPA cells resulted in considerably lower levels of reporter activation for both cavefish and zebrafish factors (Figure 7D). These results support the notion that the amino acid sequence differences in the cavefish PAR-bZip factors do not account for the loss of light and ROS-induced D-box mediated *hebp2* transcription in *P. andruzzii*. Instead, they implicate the loss of function of distinct regulatory cofactors in cavefish, which thereby attenuate PAR-bZip transcription factor-mediated activation in response to light and ROS.

## 4. Discussion

Sunlight is a powerful environmental factor for cells, influencing various physiological processes directly or indirectly by elevating levels of ROS. Previous studies in fish cells have linked its effects on the circadian clock and DNA repair systems to transcriptional activation via D-box enhancer elements found in the promoters of light-responsive genes. However, the general effects of visible light exposure and ROS on cell physiology remain incompletely understood. Here, we identified a group of light- and ROS-responsive genes associated with heme metabolism and transport, as well as mitochondrial structure and function in zebrafish cells and embryos. We demonstrate that the expression of these genes is activated via the D-box enhancer element. Furthermore, we tested the functionality of the PAR-bZip factors, showing that they are able to activate the expression of a minimal promoter reporter construct containing the D-box found in the *hebp2* gene promoter. Importantly, these genes are not upregulated by light exposure or treatment with ROS in cells of the blind Somalian cavefish *P. andruzzii*. Cavefish homologs of the PAR-bZip factors, which share significant amino acid sequence similarity with their zebrafish counterparts, also activate transcription via the D-box enhancer in zebrafish PAC-2 cells. However, both the zebrafish and cavefish transcription factors induce lower levels of reporter gene expression in the context of cavefish EPA cells. Thus, it seems unlikely that the amino acid sequence differences in the cavefish PAR-bZip factors account for the loss of light and ROS-induced D-box mediated *hebp2* transcription in *P. andruzzii*. Instead, our results imply that the function of distinct regulatory cofactors that target the PAR-bZip transcription factors is significantly attenuated in cavefish cells.

### 4.1. Role of Mitochondrial and Heme Genes

Our transcriptomic analysis has revealed significant upregulation of genes associated with mitochondrial structure and function, potentially leading to alterations in permeability, ATP output, and overall mitochondrial function with general consequences for energy metabolism. Considering the key importance of mitochondria and electron transport chain enzymes in mediating blue light-induced apoptosis [18,19], these factors could be part of an adaptive and protective mechanism counteracting the challenges posed by sunlight exposure, preserving mitochondrial integrity, and promoting cellular energy production.

The upregulation of heme-related genes further underscores the multifaceted cellular response to light. Heme is synthesised predominantly in the mitochondria and serves as a crucial cofactor for numerous enzymes within and outside this organelle [54,55,56]. It plays a pivotal role in managing oxidative stress, as a prosthetic group for various antioxidant enzymes that break down ROS molecules [2,57]. Heme is also involved in xenobiotic metabolism by serving as a cofactor for cytochrome P450 enzymes, which oxidize compounds to facilitate their excretion [58,59]. In addition, mouse knockouts of the three PAR-bZip factors have revealed a role in mediating the expression of genes involved in the metabolism of xenobiotics [39]. Furthermore, circadian rhythms of drug metabolism have been extensively documented [60,61]. Therefore, several lines of evidence support an important role for sunlight exposure in heme homeostasis both via transcriptional regulation of crucial enzymes and via affecting heme transport and metabolism. Given the loss of light and ROS-induced expression of mitochondrial and heme-related genes in *P. andruzzii* cells, it is tempting to speculate that this cavefish may consequently exhibit increased sensitivity to acute sunlight exposure, with a potentially corresponding increase in cell death. Alternatively, these regulatory differences might reflect an adaptive strategy to survive the extreme, perpetually dark cave environment. Clearly, additional studies will be required to evaluate the functional significance of these cavefish-specific changes in gene regulation networks.

### 4.2. Mechanism of D-Box Regulation

In this study, we identified functional D-box and E-box sequences in the promoters of 70% zebrafish light-regulated genes related to mitochondrial function and heme metabolism. Promoter fragments of *hebp2*, *abcb6a*, and *soul5* containing these enhancers were shown to drive light-inducible transcriptional activation. The E-box enhancer serves as the binding site for the Clock-Bmal heterodimer and thereby typically acts as a regulatory target for the core machinery of the circadian clock [62]. It is therefore surprising that none of the genes of this functional class showed evidence for circadian rhythmicity of expression under constant darkness conditions. However, other members of the bHLH PAS domain family of transcription factors may also bind to E-box-like enhancers [63]. Therefore, potentially, non-clock-regulated factors may occupy these sites and enable regulation via the D-box. The D-box-binding sequence was initially described as the canonical sequence 5′–RTTAYGTAAY–3′ (R is A or G, and Y is C or T) [30], but subsequent research has broadened the scope of D-box sequences. In the present study, one of the identified D-box sequences of the *hebp2* promoter is canonical (5′–GTGATGTAAC–3′), while the other two represent variants of this sequence (5′–GTTACTTAAC–3′ and 5′–GTTATTTAAG–3′). Interestingly, the PAR-bZip factors exhibited differential preference for *hebp2* and *cry1a* light-responsive regions. The strongest activators for *hebp2* were the TEF and HLF factors, while HLFs and DBP-2 preferentially activated *cry1a* [45]. This variability potentially highlights promoter-specific differences in the regulatory role of D-boxes. In addition, heterodimerization, post-translational modification, and other regulatory mechanisms may influence the function of these transcription factors. Further studies, such as studying knockout models for individual PAR-bZip factors, will be required to dissect the specific roles of these factors in D-box-mediated transcription in response to sunlight and ROS.

### 4.3. Evolution of Light-Induced Gene Expression

Comparing the transcriptional response to light in zebrafish with that in the Somalian blind cavefish *P. andruzzii* is a valuable comparative system for elucidating the underlying light- and ROS-regulated mechanisms, as well as understanding how they have been shaped by evolution in constant darkness [4,23,24,26]. In the present study, we reveal that zebrafish show a more robust and sustained transcriptional response to light compared to cavefish. Only a few cavefish genes have retained light-inducible expression, notably *abcb6a* and *ddb2*. We have previously demonstrated the necessity of an E2F binding site cooperating with a D-box enhancer for the induction of *ddb2* gene expression in response to visible light, UV, and ROS exposure in cavefish cells, and it is tempting to speculate that a similar mechanism operates for *abcb6a* [24]. Given the retention of functional D-box sequences in the promoters of clock and DNA repair genes in *P. andruzzii*, it is tempting to speculate that altered functionality of the cavefish PAR-bZip factors contributes to this adaptation. However, the C-terminal truncation mutations that we previously documented in a subset of opsin, core clock genes, and DNA repair genes from *P. andruzzii* [4,26] are absent from the PAR-bZip factor genes. Furthermore, the cavefish TEF and HLF homologs robustly activate transcription from the *hebp2* promoter in zebrafish cells, but not in cavefish cells. These results point to the presence of additional light and ROS-regulated cofactors, acting upstream of the PAR-bZip transcription factors, which exhibit an attenuated function in *P. andruzzii*. It is also conceivable that *P. andruzzii* carries loss-of-function mutations affecting ROS-responsive epigenetic factors, which may modulate D-box enhancer function by altering chromatin state, DNA methylation, or transcription factor accessibility, thereby controlling gene expression in a light-dependent manner [64]. During vertebrate evolution, major changes have occurred in the regulation and function of D-box binding transcription factors. Specifically, while in mammals, D-box-mediated transcriptional regulation operates in the context of clock output pathways, in fish, D-boxes serve as targets for light input pathways for the clock. Therefore, from a more general perspective, these results point to remarkable plasticity in the function of D-box-regulated transcription over the course of vertebrate evolution.

## 5. Conclusions

In the present study, the D-box enhancer element emerges as part of a broader regulatory mechanism that is not restricted to circadian clock entrainment and DNA repair. Our results point to the D-box playing a central role in the modulation of light, ROS, and UV-mediated gene expression. We reveal the multifaceted role of light-regulated transcription via the D-box in influencing cellular energy dynamics, oxidative stress response, and detoxification in fish, indicating that light exposure also influences cellular and mitochondrial metabolic function and efficiency. Furthermore, our work provides new insight into the evolutionary adaptations made by cave-dwelling organisms during evolution in their extreme aphotic environments. These include mutations that affect modulatory cofactors of the D-box-binding transcription factors and thereby elicit a global attenuation of the transcriptional response to sunlight exposure.

## Figures and Tables

**Table 1:** Alignment scores between zebrafish and cavefish PAR-bZip factors. Sequences were aligned with the EXPASY protein alignment tool (https://web.expasy.org/sim/ (accessed on 19th April 2024)).

Protein	Amino Acid Similarity	Score	Gap Frequency
TEF-1	82.1%	1261	1.0%
TEF-2	77.4%	1147	3.2%
DBP-1	93.7%	1776	1.4%
DBP-2	93.3%	1805	1.1%
HLF-1	86.7%	1303	2.7%
HLF-2	87.7%	1340	3.3%
Nfil3-1a	85.5%	1962	2.2%
Nfil3-2a	77.2%	2023	2.2%
Nfil3-2a_mut	74.2%	1253	2.9%
Nfil3-3a	56.6%	662	10.4%
Nfil3-1b	82.0%	1119	0.0%
Nfil3-2b	83.9%	2450	3.7%
Nfil3-3b	81.2%	1406	3.4%

## Data Availability

The data described in this manuscript are available from the corresponding authors upon reasonable request (nicholas.foulkes@kit.edu and daniela.vallone@kit.edu). RNA-sequencing data are available at GEO (GSE290582).

## Supplementary Materials

The following supporting information can be downloaded at: https://www.mdpi.com/article/10.3390/antiox14101151/s1, Tables and Figures: PDF file containing Table S1: All primers used. Table S2: Accession numbers. Figure S1: Representative RT-qPCR graphics for gene induction by H2O2. Figure S2: Western blot. Supplementary Dataset S1: Excel file containing: RNAseq standard ZF GOIs; enhancers—clover results; data and analysis for Figure 2, Figure 3 and Figure 4; statistical analysis for Figure 2, Figure 3 and Figure 4; data and statistical analysis for Figure 6; data and statistical analysis for Figure 7; data analysis for Figure S2. Supplementary Dataset S2: Original Western blot images.
