# Supplementary material for: Light-Induced Transcription in Zebrafish Targets Mitochondrial Function and Heme Metabolism
Source: Antioxidants (Basel). 2025 Sep 23;14(10):1151. doi: 10.3390/antiox14101151 (PMC12561580; doi:10.3390/antiox14101151)
Supplement: Supplementary file 1 [file antioxidants-14-01151-s001.zip › Supp.Dataset_2.pdf]

Original blots presented in Figure S2

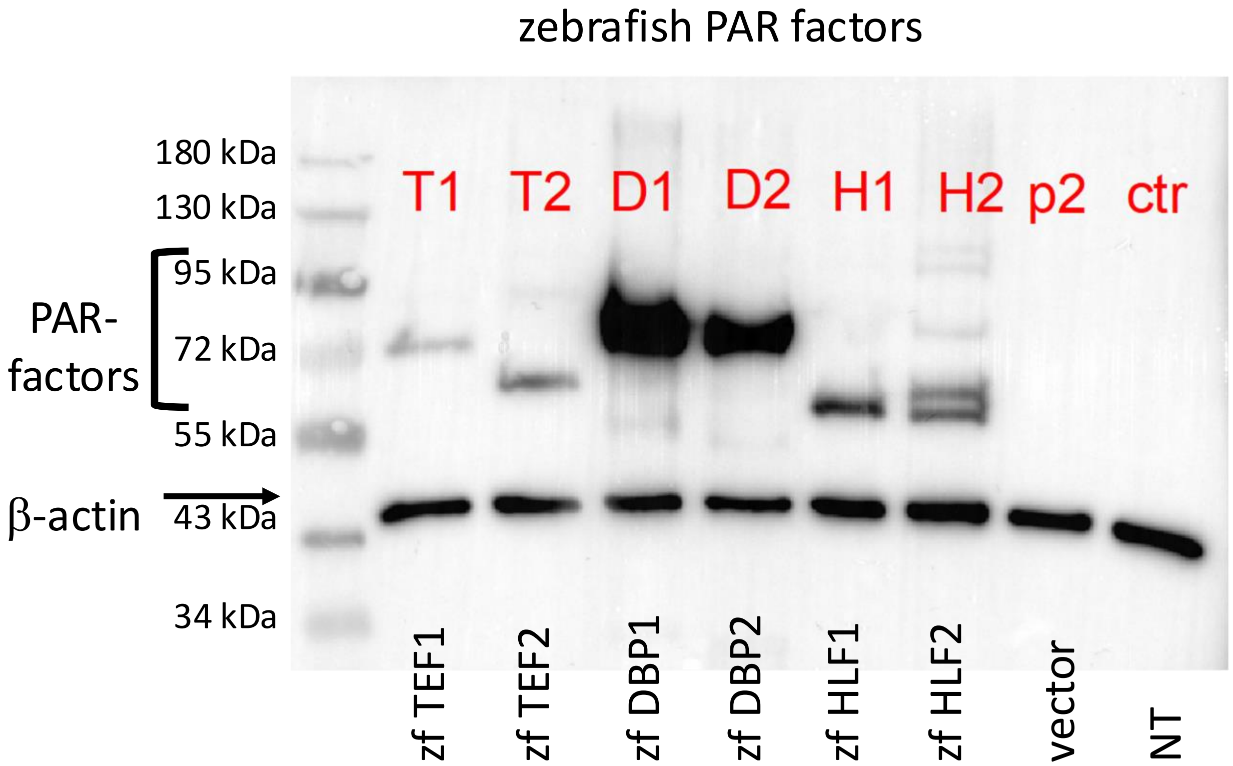

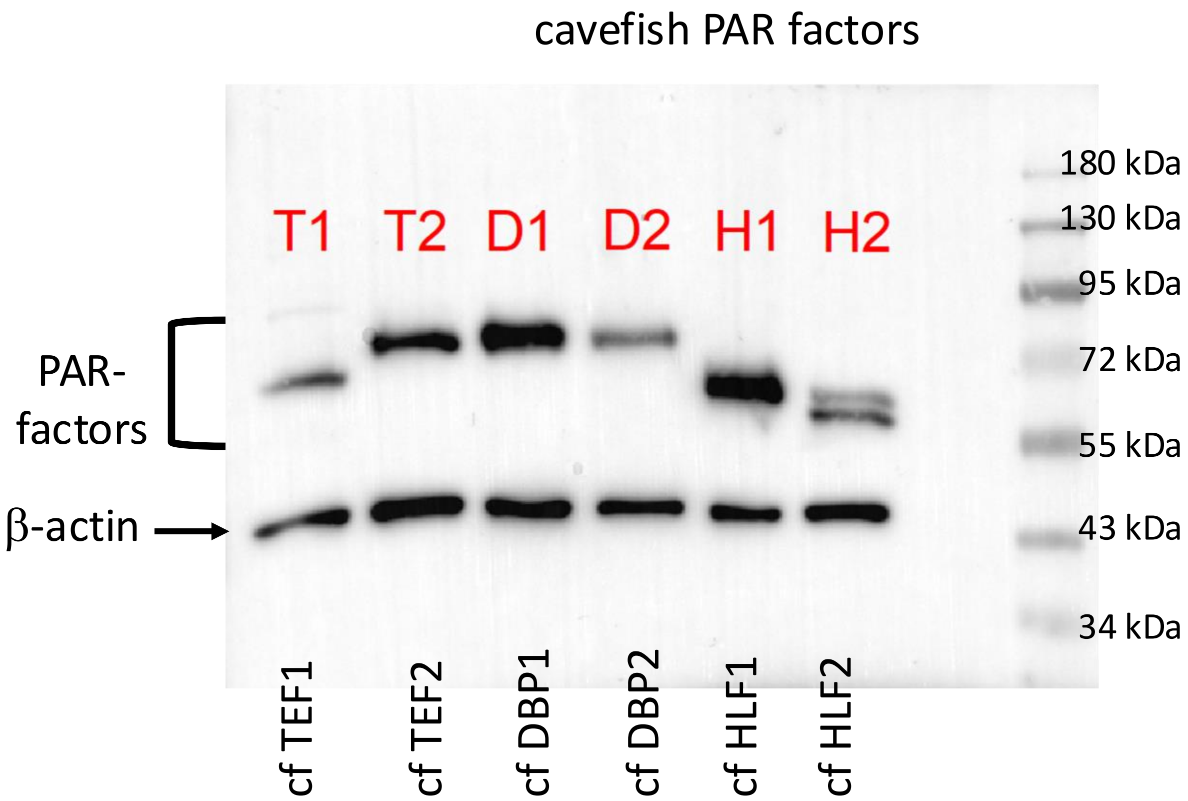

PAC-2 cells in 6-well plates were transfected with 1μg of the zebrafish and cavefish PAR-bZip expression vectors and kept in darkness for 48 hours before lysis with Passive Lysis Buffer (Promega). Samples were run on 10% polyacrylamide-SDS gels together with the Color Prestained Protein Standard, Broad Range (10-250 kDa, New England BioLabs) and blotted onto Immobilon®-P PVDF membrane (Merck Millipore). Mouse anti-Myc tag antibody (1;1000, Sigma) and goat anti-mouse polyclonal antibody (1:7500, Sigma) were used to detect protein expression. Β-actin was also detected as loading control. The identity of the samples is indicated below with NT (ctr) representing a non transfected protein extract and vector (p2) representing extract from cell transfected with the vector alone.
