# Supplementary material for: Light-Induced Transcription in Zebrafish Targets Mitochondrial Function and Heme Metabolism
Source: Antioxidants (Basel). 2025 Sep 23;14(10):1151. doi: 10.3390/antiox14101151 (PMC12561580; doi:10.3390/antiox14101151)
Supplement: Supplementary file 1 [file antioxidants-14-01151-s001.zip › Supplementary Figures and Tables.pdf]

Supplementary Tables and Figures

Table S1

RT-qPCR amplification Primers		
**gene**	**direction**	**primer sequence 5'-3'**
zf hebp2	fw	CGCACCTACCAAGCAACAA
zf hebp2	rv	TCGTCATCTCCACCTTACTCTT
zf soul5	fw	GCCACTATGATGCTGCCAAG
zf soul5	rv	ATACACTGACGACTGCCACAT
zf abcb6a	fw	AAGACTTGAAGGTGACGCTG
zf abcb6a	rv	CCATAGCGGCTGTACCAAAT
zf 6-4phr	fw	AATGGCAAGACTCCCATGAC
zf 6-4phr	rv	GTGGCCCTAAGGATGACGTA
zf blvra	fw	CGTGGCTGGTGGTTCTGTT
zf blvra	rv	AGGCTTGTGCTGTTGAGTCA
zf slc23a2	fw	TTGATGGAGTCTTTGGCACT
zf slc23a2	rv	GATCATGCCGAATAGTGTGC
zf sdha	fw	TCAAGGATCGTGTGGATGAG
zf sdha	rv	ATAGGAACGGATTGCAGGAG
zf prdx1	fw	GTCGCTCCATTGATGAAACC
zf prdx1	rv	AGTGTCTTTTCCAGGCTTCC
zf per1b	fw	ATGTGCAGGCTGTAGATCCC
zf per1b	rv	CCGTCAGTTTCGCTTTTCTC
zf actin	fw	GATGAGGAAATCGCTGCCCT
zf actin	rv	GTCCTTCTGTCCCATGCCAA
zf c-myc	fw	GGCTAGCAACAATCACAGCA
zf c-myc	rv	ATGCACTCTGTCGCCTTCTT
cf abcb6a	fw	GAGAGAAGCAGAGAGTTGCCA
cf abcb6a	rv	CAAGAATCACATCGGCTCC
cf actin	fw	GATGAGGAAATCGCTGCCCT
cf actin	rv	GTCCTTCTGTCCCATGCCAA
cf hebp2	fw	CCGCACTTACCACACAACAA
cf hebp2	rv	CTTTCACAAGTGGGTCCAGC
**RACE PCR Primers**		
**gene**	**direction**	**primer sequence 5'-3'**
cf HLF-1	5' RACE	GATTACGCCAAGCTTGGCCAGAACGTTCTTACAGCGGCCG
cf HLF-1	3' RACE	GATTACGCCAAGCTTGAACCCCATGAAGCTGCCCTTTCACCA

cf HLF-1	5' RACE nested	GATTACGCCAAGCTTCCGGTTACTGCGATCCACCACTGAAGG
cf Nfil3-1b	5' RACE	GATTACGCCAAGCTTGCTTCACGCTTTGGCACCGCAACCT
cf Nfil3-1b	3' RACE	GATTACGCCAAGCTTCCTGCCCAAGGTGATGTTGCTGGGG
cf Nfil3-1b	5' RACE nested	GATTACGCCAAGCTTAGACGCCAAGACAGAGAGGCTTCCCAT
cf Nfil3-1b	3' RACE nested	GATTACGCCAAGCTTATGGGTGGTTTCTTCGTATCGCCACGG
cf TEF-2	5' RACE	GATTACGCCAAGCTTCAATCTGGTTCTCTTTCAGACGCCG
cf TEF-2	3' RACE	GATTACGCCAAGCTTCGGAGGAGATCGAGGTGAACGTGG
cf TEF-2	3' RACE nested	GATTACGCCAAGCTTCCCACAGATCTGGTCCTGTCCAGCGTT
cf HLF-1	fw	CGGCGGCTGAAAGAAAACC
cf HLF-1	rv	GTGTCGCGCCTCGTATTTG
**Primers for PAR and Nfil3 factors**	**insertion in pGEM-T Easy**	
**gene**	**direction**	**primer sequence 5'-3'**
cf HLF-1	fw	GCGATGGAGAAGATGGAGAAGA
cf HLF-1	rv	GTTGGGGGCTCACAGAGGG
cf TEF-2	fw	GATAATATCGCGCTAATGATGCCC
cf TEF-2	rv	GAAGCGTGTCCCTCACAGTGAG
cf Nfil3-1b	fw	TGAAAATGGAGTCTGCTTTC
cf Nfil3-1b	rv	TCAATCTGACAAGTACACTGG
cf Nfil3-2a	fw	AGGAGCAATGGAAAGTTTGAGC
cf Nfil3-2a	rv	GGTTTCTTGGCGTTGTTGCT
cf Nfil3-2a	fw	AGCACAATTTTGAGTCAGGT
cf Nfil3-2a	rv	TTGTTTAGTCATGTCTCTTTTTACA
cf Nfil3-2b	fw	TCTTTGGAGGAAAAAGCAGAAGC
cf Nfil3-2b	rv	ACCTGCTCCATGTCCTCAAC
cf Nfil3-2b	fw	ACCCCAAAGAAGCGTCATCC
cf Nfil3-2b	rv	TCAATAGGATGGAAAGGTGACA
cf Nfil3-3a	fw	CACACACTAACTCAAAGCATGAAGG
cf Nfil3-3a	rv	GTGGGACAGATGATTTCAGTTCAC
**PAR and Nfil3 factors insertion in pCS2-MTK**		
**gene**	**direction**	**primer sequence 5'-3'**
cf DBP-1	fw	CCTCCAAGCCAATTTCTCAG
cf DBP-1	rv	CCTCAAAGATCTCCGTGGCGG
cf DBP-2	fw	CGCGAATTCACTAGTGATTTGTTGGCCAGG
cf HLF-2	fw	TAACGAATTCTTGTCTAGACAGCTC
cf TEF-1	fw	GGGAATTCGATTCTCGGACAAACTTGGAC
cf TEF-2	fw	CTAGTGAATTCATCGCGCTATTGATGCCC

cf Nfil3-1a	fw	CGCGAATTCACTAGTGATTTGCAAGCCA
cf Nfil3-1b	fw	GAATTCGATTTGAAACTCGAGTCGCTTTC
cf Nfil3-1b	rv	GCGAATTCTCGAGTGATTTCAATCTGACAA
cf Nfil3-2a	fw	GAATTCGATTAGGCCTCATTGGAAAGTTTG
cf Nfil3-2b	fw	CTTAAAAGGCCTATTTGGAAAGCCTAA
cf Nfil3-3a	fw	CACACACTAACTCGAGGCTTGAAGGACC
cf Nfil3-3a	rv	GATTTGTGGCTCGAGATGATTTCAGTTCAC
cf Nfil3-3b	fw	CGCGGGAATTCGATTTGTCTTTCACCA
**Sequence Primers**		
Sp6		TATTTAGGTGACACTATA
T7		TAATACGACTCACTATAGGG
**Promoter insertion primers**		
**Promoter**	**Direction**	**Primer 5'-3'**
zf abcb6a	fw	GCATAAATAGATCAAAAAGCTTGATAGGTAACAG
zf abcb6a	rv	GTCACCTTCAAGCTTTCGAGTGC
zf hebp2	fw	GTTTTTTAAAGAAGGAAGCTTGTGTAATTTTC
zf hebp2	rv	CTGTTGACAAGCTTAAAAGCAGAAAGC
zf soul5	fw	CAGTCTGTAGTCAAAAGCTTGTGAATG
zf soul5	rv	GAATTTTGTAAGCTTCAGGCTGAGCA
zf hebp2	fw	TCGTCACAACCAATCGGTACCTTC
zf hebp2	rv	CGCTGTTAAATAACGCTAGCGCTTAAG
zf soul5	fw	AAGCAGGTACC GTGATTTTTTTCTTTTGTTAC
zf soul5	rv	GCTTAGCTAGC AAGAGAGAGGAAACGTGTG
**Hebp2-Luc promoter mutations**		
**Promoter**	**Direction**	**Primer 5'-3'**
zf hebp2 D-box 1 mut	fw	TCGCATGAAAACACGGAAGTAGTCCTGTC
zf hebp2 D-box 1 mut	rv	CGTCGCTCCCATGCGAAGGCTCCG
zf hebp2 D-box 2 mut	fw	CTCCGCGTAGAGTTATTTAACAGCG
zf hebp2 D-box 2 mut 2	fw	CTCCGCGTAGAGCGAGTACTCAG
zf hebp2 D-box 2 mut	rv	CTCGCGCTGACAGGACTACTTCCG
zf hebp2 D-box 3 mut	fw	TACTCAGCGAAACTGTATATTCTC
zf hebp2 D-box 3 mut 2	fw	TACTCAGCGAAACTGTATATTCTCTATCTAAGCGTTATAACAC
zf hebp2 D-box 3 mut	rv	CTCGCTCTACGCTTAAGTAACGC
zf hebp2 D-box 3 mut 2	rv	CTCGCTCTACGCGGAGCTCGCGC

Table S2

Accession numbers RNAseq	gene	species			BankIt2824219 submission – cavefish PAR-bZip and Nfil3 factors	
ENSDARG00000011583	cry1a	zebrafish			**Sequence**	**GenBank number**
ENSDARG00000016721	sdha	zebrafish			cf TEF-1	PP750800
ENSDARG00000017365	slc23a2	zebrafish			cf TEF-2	PP750801
ENSDARG00000019498	6-4 phr	zebrafish			cf DBP-1	PP750802
ENSDARG00000034503	per2	zebrafish			cf DBP-1	PP750803
ENSDARG00000042630	hebp2	zebrafish			cf DBP-2	PP750804
ENSDARG00000058734	prdx1	zebrafish			cf HLF-2	PP750805
ENSDARG00000059857	blvra	zebrafish			cf Nfil3-1a	PP750806
ENSDARG00000063297	abcb6a	zebrafish			cf Nfil3-2a	PP750807
ENSDARG00000075015	soul5	zebrafish			cf Nfil3-2a_mut	PP750808
					cf Nfil3-3a	PP750809
**Trinity transcripts (*de novo* analysis)**	**gene**	**species**			cf Nfil3-1b	PP750810
TRINITY_DN112_c16_g1	abcb6a	zebrafish			cf Nfil3-2b	PP750811
TRINITY_DN2072_c1_g1	soul5	zebrafish			cf Nfil3-3b	PP750812
TRINITY_DN5811_c0_g1	per2	zebrafish				
TRINITY_DN717_c3_g1	hebp2	zebrafish				
TRINITY_DN14099_c0_g1	hebp2	cavefish				
TRINITY_DN32848_c0_g1	per2	cavefish				
TRINITY_DN47926_c0_g1	abcb6a	cavefish				

Figure S1

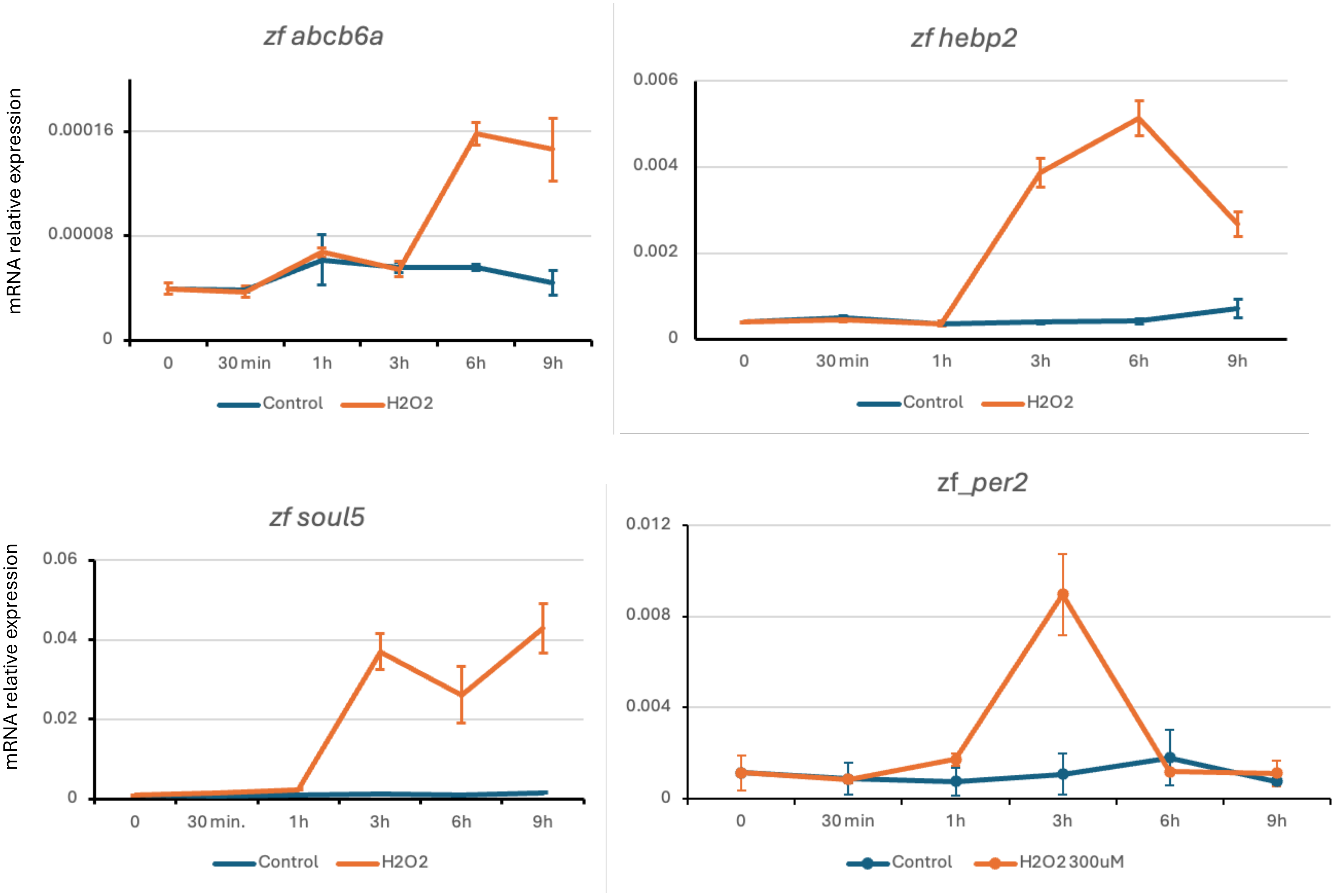

Figure S1. mRNA expression in PAC2 cells treated with H_2O_2. Representative graphics (from 1 biological replicate) of RT-qPCR results for the expression of *abcb6a*, hebp2, soul5 and per2 in PAC2 cells treated in darkness with 300 µM of H2O2 (red traces) or with PBS (control, dark blue traces) as described in Figure 4B. In the X-axis are reported the hours of treatment and on the Y-axis the mRNA relative expression. Data were analysed according to the $2^{-\Delta\Delta CT}$ method and expression of *β-actin* was used for normalization. Standard deviation from n=3 replicates is reported. Note that the changes in mRNA expression in the control samples showed no significant changes during the 9 hours of incubation in constant darkness.

Figure S2

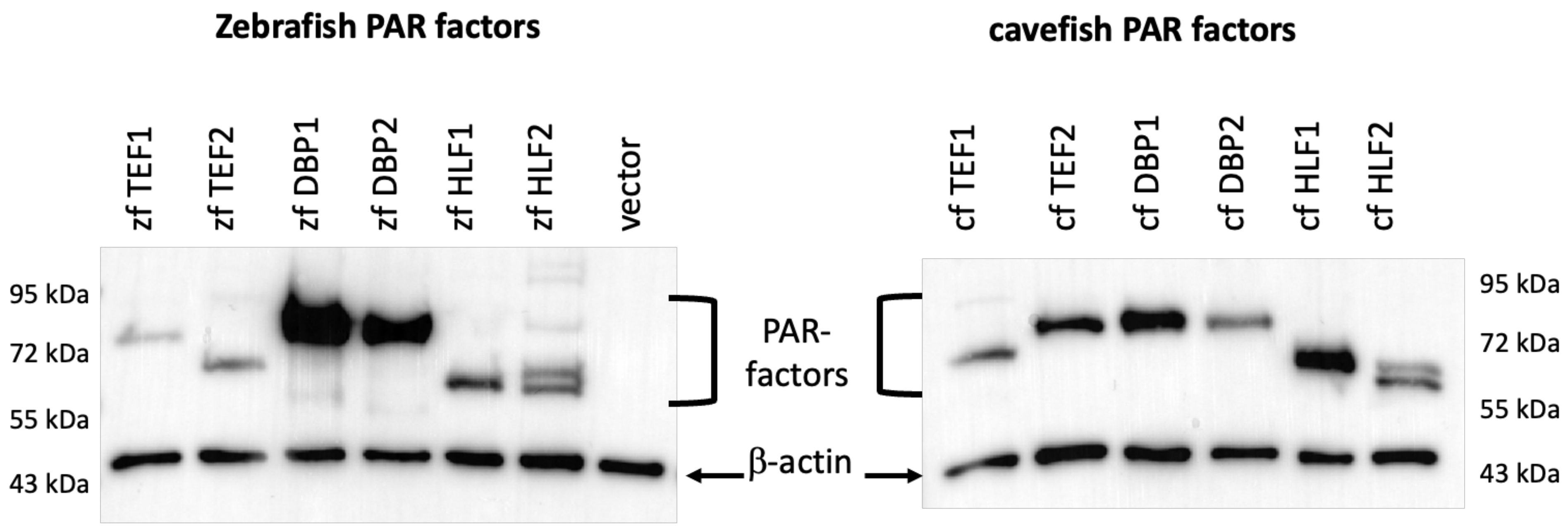

Figure S2. Western blot of overexpression of zebrafish and cavefish PAR-bZip factors. PAC2 cells were transiently transfected with 1µg of the expression vectors, which contain a 5x MYC tag at the N-terminal of the proteins. Β-actin was also detected as loading control. The identity of the samples is indicated above with vector representing extract from cell transfected with the vector alone. Samples were run on 10% polyacrylamide-SDS gels together with the Color Prestained Protein Standard, Broad Range (10-250 kDa, New England BioLabs). For original western blot image see Supplementary Dataset_2
